# A Rare Case of Simultaneous Evans Syndrome and Primary Antiphospholipid Syndrome

**DOI:** 10.7759/cureus.6845

**Published:** 2020-02-01

**Authors:** Bhamini P Patel, John Jakob

**Affiliations:** 1 Medicine, Northeast Ohio Medical University, Rootstown, USA; 2 Hematology/Oncology, Summa Akron City Hospital, Summa Health System, Akron, USA

**Keywords:** antiphospholipid syndrome, thrombocytopenia, evans syndrome

## Abstract

Evans Syndrome (ES) is a rare autoimmune disorder that presents with simultaneous or sequential development of autoimmune hemolytic anemia (AIHA), thrombocytopenia, and/or neutropenia. This disease may occur in conjunction with other autoimmune disorders. Primary antiphospholipid syndrome (APS) is a disorder characterized by thrombosis, which can cause life-threatening complications such as fetal demise, strokes, or deep vein thrombosis.

A 67-year-old male with type 2 diabetes mellitus, hypertension, and renal insufficiency presented with concomitant ES and APS. His hematological abnormalities began in 2013 after a deep vein thrombosis of the left lower extremity led to a diagnosis of APS and was started on chronic warfarin. In 2014, he was found to have immune thrombocytopenia (ITP) with relapses the following year. Several months later, he was hospitalized and diagnosed with AIHA. In the setting of his previous episodes of ITP and current AIHA, the diagnosis of ES was made. The initial treatment was 100 mg prednisone taper, but rituximab was required to make complete platelet recovery.

The severe deterioration and rapid recovery with proper treatment of the patient highlights the importance of a timely diagnosis of ES. Mild thrombocytopenia can be associated with APS; however; severe thrombocytopenia may warrant further investigation for other possible causes. Maintaining ES on the differential diagnosis of patients with APS and thrombocytopenia could enhance health outcomes.

## Introduction

Evans syndrome (ES) is a rare autoimmune disorder that presents with simultaneous or sequential development of autoimmune hemolytic anemia (AIHA), thrombocytopenia, or immune-mediated neutropenia [1].

Primary antiphospholipid syndrome (APS) is an acquired antibody-mediated disorder characterized by thrombosis causing recurrent fetal demise, stroke, deep vein thrombosis (DVT), or other arterial thrombotic events. Primary APS can also be associated with thrombocytopenia and hemolytic anemia, findings required for a diagnosis of ES [2]. In this case, a diagnosis of ES was initially masked by confirmed APS and thrombocytopenia due to the overlapping presentations of the two syndromes. 

## Case presentation

A 67-year-old Caucasian male with a past medical history of type 2 diabetes mellitus, hypertension, and chronic kidney disease presented with unprovoked DVT of the left lower extremity in March 2013. Coagulation work-up revealed elevated beta-2 glycoprotein I (β2GPI) IgM and cardiolipin IgM antibody titers greater than 99th percentile and repeat titers confirmed the diagnosis of primary APS, and the patient was started on lifelong warfarin.

The patient’s hematological status was further complicated with a diagnosis of immune thrombocytopenia (ITP) after a platelet count of 7 x 10^9^/L and a right lower extremity DVT in 2014; a 40 mg prednisone taper resolved his symptoms. However, relapse occurred in August 2015 with a platelet count of 48 x 10^9^/L. Re-initiation of prolonged prednisone taper resulted in a gradual return of platelets to baseline. In November 2015, the patient was admitted to the hospital for jaundice, low hemoglobin (6.0 g/dl), and laboratory confirmation of warm AIHA. Patient received four units ofpacked red blood cells, 100 mg prednisone, and two treatments of intravenous immunoglobulin therapy. AIHA in conjunction with a previous history of ITP led to a final diagnosis of ES two years after initial presentation.

A 100 mg prednisone taper was initiated, and the platelet count maintained at levels > 40 x 10^9^/L with low-dose prednisone (5-10 g) until November 2016 when worsening platelet count required a 20 mg prednisone burst. A steady platelet count of 233 x 10^9^/L was achieved after completing four doses of rituximab therapy in February 2017. One year later, the patient is doing well on warfarin with normal platelet counts and no episodes of thrombosis or anemia.

## Discussion

ES is a rare disorder that can have a potentially deadly outcome due to the rapid decline in hemoglobin and platelet count. It is a diagnosis of exclusion, requiring a history of Coombs-positive hemolytic anemia, thrombocytopenia, and/or neutropenia. Several autoimmune disorders have been known to occur in conjunction with ES including autoimmune lymphoproliferative syndrome, lupus, Sjogren's syndrome, and common variable immunodeficiency [3,4]. Additionally, HIV and HCV have been associated with the development of ES, and an infectious work-up is also warranted [5].

Primary APS is an acquired antibody-mediated disorder characterized by thrombosis occurring in the arterial or venous vasculature. The diagnosis of primary APS requires two elevated serum studies of β2GPI, lupus anticoagulant, or cardiolipin antibodies obtained 12 weeks apart [2].

ES has long been considered an incidental finding of ITP and AIHA, but recent studies suggest a more complex immune dysregulation of the humoral and cellular systems [3]. However, researchers and clinicians have followed a trial-and-error approach to treatment and management of ES.

Several therapy options are available due to the broad spectrum of presentations in ES. Corticosteroids and IVIG serve as the mainstay of therapy for patients presenting with ITP or AIHA [4]. In a retrospective study of 68 patients with ES, 83% of patients experienced a complete or partial response to corticosteroids. However, most patients required additional therapy for symptom control with only 18 patients maintaining long-term remission solely after steroid treatment, which demonstrates the need for secondary treatment options in the management of ES [1].

**Figure 1:**
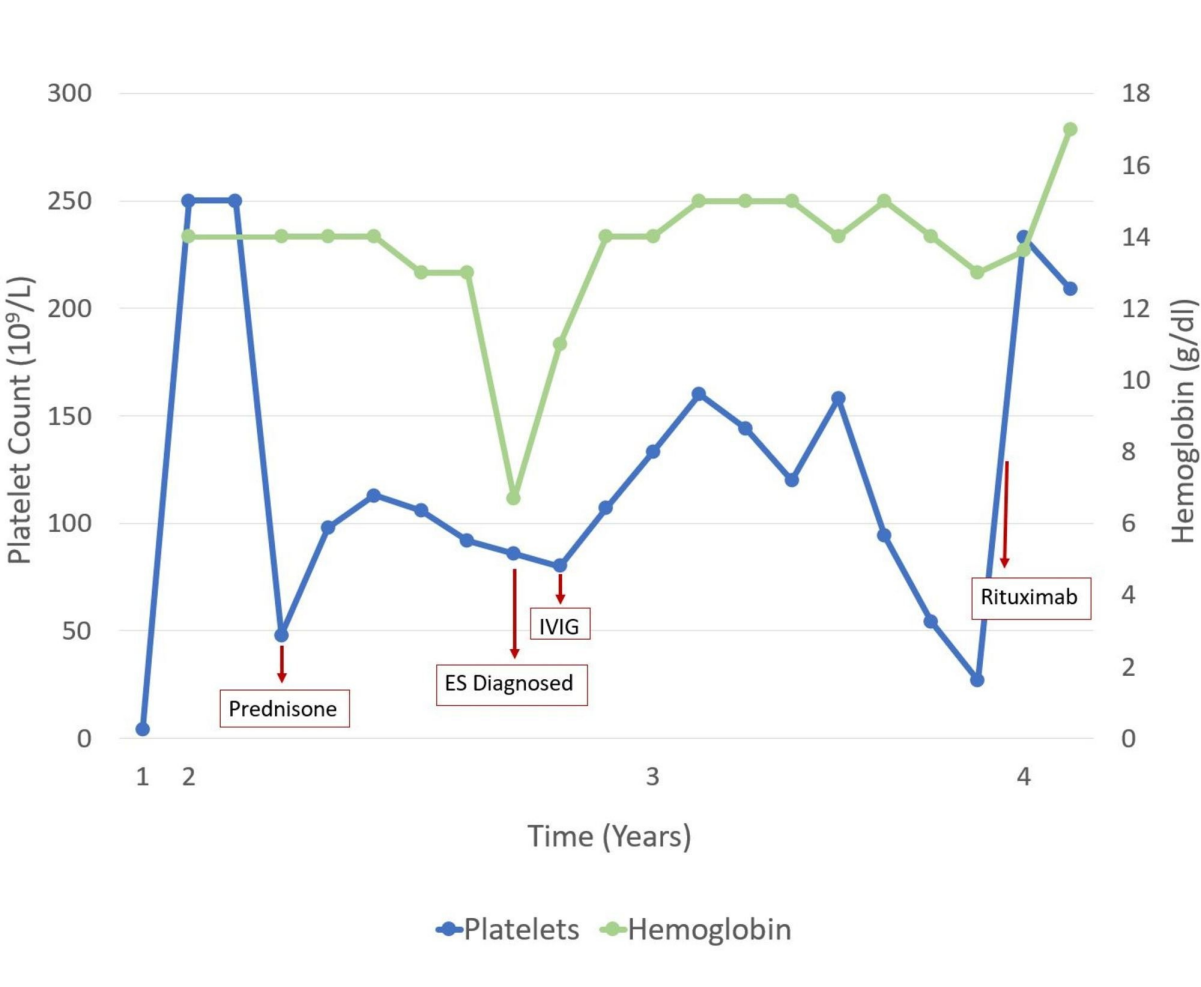
Platelet and Hemoglobin Levels in Relationship to Diagnosis and Treatment Figure represents the change in platelet count and hemoglobin levels throughout the course of three years. Various medications used in the treatment of the patient are shown to demonstrate its affect on platelet count.  Platelet count was responsive to high-dose prednisone, but levels decreased as prednisone dose was tapered. Intravenous immunoglobulin (IVIG) therapy was used twice after the initial autoimmune hemolytic anemia (AIHA) and diagnosis of Evans syndrome (ES). Finally, the use of rituximab, a monoclonal CD20 antibody, provided the greatest increase in platelet count demonstrating its efficacy in disease management.

The lack of an established treatment guideline for ES has led to the use of medications based on anecdotal evidence. Some of these medications include danazol, cyclophosphamide, hydroxychloroquine, cyclosporine, azathioprine, and dapsone. Rituximab, a CD20 antibody, has shown recent success for sustained complete response [1]. Figure 1 highlights the cellular response to the various treatment modalities by the patient. However, not all patients maintain a complete remission and often require secondary treatment. In refractory disease, splenectomy has become a common choice of treatment although success rates are lower than the 70%-75% response rate found in ITP [4,6,7]. The stark difference in success rates between ES and classic ITP or AIHA suggests varying pathology. Additionally, there are few studies looking at the long-term outcomes of patients with splenectomies.

ES is not limited to physical complications, and may affect psychological well-being. Existing literature has highlighted a relationship between autoimmune disease and psychopathology [8]. Continuous episodes of relapse and remission may create significant stress and worry for ES patients. Patients may benefit from additional inquiry into the psychological complications of a chronic autoimmune disease.

## Conclusions

Patients with primary APS may experience severe consequences. Although mild thrombocytopenia and hemolytic anemia can be associated with APS, severe thrombocytopenia may warrant further investigation for other possible causes. ES presents as thrombocytopenia or neutropenia and hemolytic anemia, which can be mistaken for common symptoms found in primary APS. However, clinicians should be cautious of these presentations as they can suggest a severe underlying pathology such as ES. Additionally, patients with β2GPI antibodies and autoimmune hemolysis are at increased risk of future thrombosis. Maintaining ES on the differential diagnosis of patients with APS and thrombocytopenia could enhance health outcomes.
